# Genome-wide analysis of WRKY gene family in the sesame genome and identification of the WRKY genes involved in responses to abiotic stresses

**DOI:** 10.1186/s12870-017-1099-y

**Published:** 2017-09-11

**Authors:** Donghua Li, Pan Liu, Jingyin Yu, Linhai Wang, Komivi Dossa, Yanxin Zhang, Rong Zhou, Xin Wei, Xiurong Zhang

**Affiliations:** 1 Oil Crops Research Institute of the Chinese Academy of Agricultural Sciences, Key Laboratory of Biology and Genetic Improvement of Oil Crops, Ministry of Agriculture, No.2 Xudong 2nd Road, Wuhan, 430062 China; 2 Centre d’Etudes Régional pour l’Amélioration de l’Adaptation à la Sécheresse (CERAAS), BP 3320 Route de Khombole, Thiès, Sénégal; 3 College of Life and Environmental Sciences, Shanghai Normal University, Shanghai, 200234 China

**Keywords:** Drought stress, Expression profiling, Sesame, Waterlogging stress, WRKY

## Abstract

**Background:**

Sesame (*Sesamum indicum* L.) is one of the world’s most important oil crops. However, it is susceptible to abiotic stresses in general, and to waterlogging and drought stresses in particular. The molecular mechanisms of abiotic stress tolerance in sesame have not yet been elucidated. The WRKY domain transcription factors play significant roles in plant growth, development, and responses to stresses. However, little is known about the number, location, structure, molecular phylogenetics, and expression of the *WRKY* genes in sesame.

**Results:**

We performed a comprehensive study of the *WRKY* gene family in sesame and identified 71 *SiWRKYs*. In total, 65 of these genes were mapped to 15 linkage groups within the sesame genome. A phylogenetic analysis was performed using a related species (*Arabidopsis thaliana*) to investigate the evolution of the sesame *WRKY* genes. Tissue expression profiles of the *WRKY* genes demonstrated that six *SiWRKY* genes were highly expressed in all organs, suggesting that these genes may be important for plant growth and organ development in sesame. Analysis of the *SiWRKY* gene expression patterns revealed that 33 and 26 *SiWRKYs* respond strongly to waterlogging and drought stresses, respectively. Changes in the expression of 12 *SiWRKY* genes were observed at different times after the waterlogging and drought treatments had begun, demonstrating that sesame gene expression patterns vary in response to abiotic stresses.

**Conclusions:**

In this study, we analyzed the WRKY family of transcription factors encoded by the sesame genome. Insight was gained into the classification, evolution, and function of the *SiWRKY* genes, revealing their putative roles in a variety of tissues. Responses to abiotic stresses in different sesame cultivars were also investigated. The results of our study provide a better understanding of the structures and functions of sesame *WRKY* genes and suggest that manipulating these *WRKYs* could enhance resistance to waterlogging and drought.

**Electronic supplementary material:**

The online version of this article (10.1186/s12870-017-1099-y) contains supplementary material, which is available to authorized users.

## Background

Sesame (*Sesamum indicum* L.) is an important, and probably the most ancient, oil crop and is grown widely in tropical and subtropical regions of the world [1]. Recently, the demand for sesame has increased, but sesame yields have been poor compared with those of other oil crops (e.g., rapeseed: 1939.3 kg/ha; soybean: 2498.5 kg/ha; and peanut: 1657.6 kg/ha). Average sesame yields were alarmingly low between 2010 and 2014, with only 576.1 kg/ha being produced in 73 countries (http://faostat.fao.org). This low yield may be attributable to a variety of factors, although abiotic stresses are certainly one of the most significant.

For sesame, the most important abiotic stresses that limit plant growth, development, and yield are drought and waterlogging. In central China, which is the major sesame production area, sesame is generally planted during the rainy season, when waterlogging is the most significant problem and can decrease sesame yields by more than 80% [2]. Sesame is also grown extensively in the tropical regions of Africa and South America. Here, drought presents a major challenge and can limit the yield from sesame by affecting the number of capsules produced by each plant [3]. Therefore, there is an urgent requirement to understand the molecular mechanisms that underlie the ability of sesame plants to tolerate both drought and waterlogging stresses.

Abiotic stress responses and gene regulation have been studied in a number of plant species, including *Arabidopsis*, rice, maize, and tomato. Several families of genes are particularly associated with significant improvements in abiotic stress tolerance, including the *WRKY*, *NAC*, and *ERF* gene families [4–6]. Numerous studies have demonstrated that *WRKY* genes are expressed strongly and rapidly in response to particular abiotic stresses, including wounding, waterlogging, drought, and salt stress [7–9]. In *Arabidopsis*, *AtWRKY30* is induced by methyl viologen, hydrogen peroxide, arsenic, drought, sodium chloride, and mannitol [10]. Nuruzzaman et al. [11] identified five *OsWRKY* genes expressed at higher levels in drought-tolerant rice compared with those in drought-susceptible rice under experimental water-deficit conditions. Overexpression of *OsWRKY47* increased both the yield and drought tolerance compared with wild-type plants [12]. Meng et al. [13] discovered that 10 selected *WRKY* genes showed differential expression patterns under waterlogging and drought stress in an apple rootstock. These observations suggest that studying *WRKY* gene families may provide valuable insights into the mechanism underlying abiotic stress tolerance in plants. Furthermore, although drought and waterlogging may primarily affect plants grown in different parts of the world, very little is known about the identity and functions of *WRKY* genes in sesame.

Over the last decade, WRKY transcription factors have become one of the most extensively studied gene families involved in regulating plant abiotic stress tolerance [14]. WRKY proteins have one or two unique DNA-binding domains that are approximately 60 amino acids (aa) in length and contain the WRKYGQK sequence followed by a C_2_H_2_ zinc-finger-like motif [15]. The DNA-binding region is designated a WRKY domain because the WRKYGQK aa sequence is completely conserved. The WRKY proteins are classified into three major groups (I–III) based on the number of WRKY domains and the pattern of zinc-finger-like motifs. Group II is further divided into five distinct subgroups (IIa–IIe) [15]. The first identified *WRKY* gene, *SPF1*, was cloned from sweet potato (*Ipomoea batatas*) 20 years ago [16]. Since then, a large number of *WRKY* genes have been identified, including 74 from *Arabidopsis thaliana* [17], 103 from *Oryza sativa* [18], 45 from *Hordeum vulgare* [19], 119 from *Zea mays* [20], and many more from other plant species [21–23].

Sequencing of the entire sesame genome, and annotation of its 24,148 putative genes, provides an opportunity to identify all the sesame *WRKY* genes [24–26]. In this study, 71 *WRKY* genes were identified from the sesame genome and analyses of their structure, phylogeny, chromosomal distribution and duplication, conserved motifs, and stimulation in response to waterlogging and drought were performed. The results provide insights into the evolution of the sesame *WRKYs* and their functions in abiotic stress responses. The identification and characterization of these *WRKY* genes may provide opportunities to improve the stress tolerance of sesame.

## Results

### Identification of *WRKY* family genes in sesame

All *Arabidopsis* WRKY protein sequences were used as queries for the Basic Local Alignment Search Tool (BLAST) to identify sesame WRKY proteins. In total, 61 putative *WRKY* genes were identified and predicted protein sequences without a WRKY domain were excluded. A Hidden Markov Model (HMM) search was also performed against the sesame protein database using the WRKY-domain PF03106. An additional 10 protein sequences containing the complete WRKY domain were identified. In total, 71 WRKY proteins were identified in the sesame genome (Table 1).

**Table 1:** Informations of *SiWRKY* genes

Gene symbol	Gene locus	Linkage group	Peptide length	pI	MW	Group
*SiWRKY1*	SIN_1000785	scaffold00233	186	7.26	20.85	NG
*SiWRKY2*	SIN_1001523	scaffold00164	302	5.63	33.94	IIe
*SiWRKY3*	SIN_1001786	LG12	300	6.30	33.82	IIc
*SiWRKY4*	SIN_1001880	LG04	760	6.28	84.34	I
*SiWRKY5*	SIN_1002759	scaffold00124	355	5.95	40.01	III
*SiWRKY6*	SIN_1002960	scaffold00120	122	9.74	14.44	IIc
*SiWRKY7*	SIN_1003153	LG14	728	5.96	78.03	I
*SiWRKY8*	SIN_1003599	scaffold00109	367	No	No	IIc
*SiWRKY9*	SIN_1003920	LG05	540	6.27	60.10	I
*SiWRKY10*	SIN_1003975	LG13	176	9.26	20.25	IIc
*SiWRKY11*	SIN_1004161	LG15	187	9.47	21.25	IIc
*SiWRKY12*	SIN_1004874	LG15	291	9.72	31.60	IId
*SiWRKY13*	SIN_1005422	LG02	297	9.21	32.27	IId
*SiWRKY14*	SIN_1005676	LG11	315	4.86	34.41	IIe
*SiWRKY15*	SIN_1005706	LG11	201	5.13	22.75	III
*SiWRKY16*	SIN_1006024	LG08	561	6.14	60.65	IIb
*SiWRKY17*	SIN_1006129	LG07	497	5.77	54.61	NG
*SiWRKY18*	SIN_1006550	LG08	513	8.28	56.11	I
*SiWRKY19*	SIN_1006749	LG12	462	5.38	49.64	IIe
*SiWRKY20*	SIN_1006978	LG12	573	6.44	61.81	IIb
*SiWRKY21*	SIN_1007987	LG15	479	6.06	52.05	IIb
*SiWRKY22*	SIN_1008040	LG15	511	8.04	55.40	I
*SiWRKY23*	SIN_1009399	scaffold00057	626	8.28	69.68	I
*SiWRKY24*	SIN_1009643	LG06	345	9.55	38.85	IId
*SiWRKY25*	SIN_1009858	LG11	151	6.23	17.23	NG
*SiWRKY26*	SIN_1010783	LG01	296	8.67	32.83	IIa
*SiWRKY27*	SIN_1010982	LG11	327	6.43	36.72	III
*SiWRKY28*	SIN_1011023	LG11	526	5.79	58.18	I
*SiWRKY29*	SIN_1011192	LG11	1141	8.49	125.94	IIc
*SiWRKY30*	SIN_1011284	LG11	452	9.10	49.38	I
*SiWRKY31*	SIN_1011416	LG03	346	9.70	37.75	IId
*SiWRKY32*	SIN_1012054	LG04	316	5.27	35.60	III
*SiWRKY33*	SIN_1012055	LG04	337	5.70	37.18	III
*SiWRKY34*	SIN_1012623	LG06	281	4.83	31.76	NG
*SiWRKY35*	SIN_1012631	LG06	293	5.26	32.48	IIc
*SiWRKY36*	SIN_1012891	LG06	336	9.70	37.73	IId
*SiWRKY37*	SIN_1014111	LG01	332	6.31	36.70	IIe
*SiWRKY38*	SIN_1014143	LG01	350	5.05	39.25	III
*SiWRKY39*	SIN_1014268	LG12	723	6.13	78.07	I
*SiWRKY40*	SIN_1014366	LG12	584	6.52	63.04	I
*SiWRKY41*	SIN_1014422	LG12	397	7.21	43.06	IIb
*SiWRKY42*	SIN_1015494	LG06	187	8.55	21.30	IIc
*SiWRKY43*	SIN_1015496	LG06	338	5.51	37.95	IIe
*SiWRKY44*	SIN_1016166	LG03	372	6.17	41.48	IIc
*SiWRKY45*	SIN_1016382	LG03	602	5.62	64.69	IIb
*SiWRKY46*	SIN_1016491	LG04	365	4.81	39.49	IIe
*SiWRKY47*	SIN_1016829	LG16	591	6.08	62.98	IIb
*SiWRKY48*	SIN_1017975	LG01	330	5.30	37.22	III
*SiWRKY49*	SIN_1017989	LG02	152	9.30	17.70	IIc
*SiWRKY50*	SIN_1018215	LG02	564	8.06	61.82	I
*SiWRKY51*	SIN_1018227	LG02	334	5.59	35.28	IIc
*SiWRKY52*	SIN_1018859	LG04	316	6.00	34.30	IIc
*SiWRKY53*	SIN_1019334	LG14	316	7.74	35.63	IIc
*SiWRKY54*	SIN_1019555	LG08	444	No	No	IIe
*SiWRKY55*	SIN_1019627	LG08	312	6.11	34.81	IIc
*SiWRKY56*	SIN_1019661	LG08	285	4.81	32.22	IIe
*SiWRKY57*	SIN_1019937	LG05	504	6.16	54.40	IIb
*SiWRKY58*	SIN_1020605	LG06	346	9.65	36.99	IId
*SiWRKY59*	SIN_1020883	LG06	160	7.76	18.49	IIc
*SiWRKY60*	SIN_1021497	LG01	168	6.52	18.90	IIc
*SiWRKY61*	SIN_1021618	LG01	268	8.92	29.50	IIa
*SiWRKY62*	SIN_1021622	LG01	255	8.81	28.19	IIa
*SiWRKY63*	SIN_1021665	LG01	334	9.54	36.05	IId
*SiWRKY64*	SIN_1021953	LG03	308	9.30	34.42	IIa
*SiWRKY65*	SIN_1022426	LG06	341	6.83	38.16	IIc
*SiWRKY66*	SIN_1022971	LG08	493	8.37	54.34	IIb
*SiWRKY67*	SIN_1023226	LG06	542	8.08	59.73	I
*SiWRKY68*	SIN_1026059	LG10	493	6.58	54.73	IIb
*SiWRKY69*	SIN_1026464	LG08	162	8.89	18.95	IIc
*SiWRKY70*	SIN_1026809	LG08	365	8.85	39.90	IIb
*SiWRKY71*	SIN_1026948	LG08	564	6.19	60.99	IIb

*pI* proteins’ isoelectric point, *MW* molecular weight

The 71 sesame WRKY proteins ranged from 122 (*SiWRKY6*) to 1141 (*SiWRKY29*) aa in length, with an average length of approximately 390 aa. The molecular weights (MWs) ranged from 14.44 kDa (*SiWRKY6*) to 125.94 kDa (*SiWRKY29*). The isoelectric points (pIs) of the WKRY proteins ranged from 4.81 (*SiWRKY46* and *SiWRKY56*) to 9.74 (*SiWRKY6*), with 39 pIs <7 and the remaining pIs >7 (Table 1). Similar observations were made in Chinese cabbage [27], which has 56 *BcWRKYs* with pIs ranging from 4.69 to 10.45 and MWs ranging from 20.44 kDa to 119.84 kDa.

### Chromosomal locations of and duplication events of the *SiWRKY* genes

**Fig. 1:**
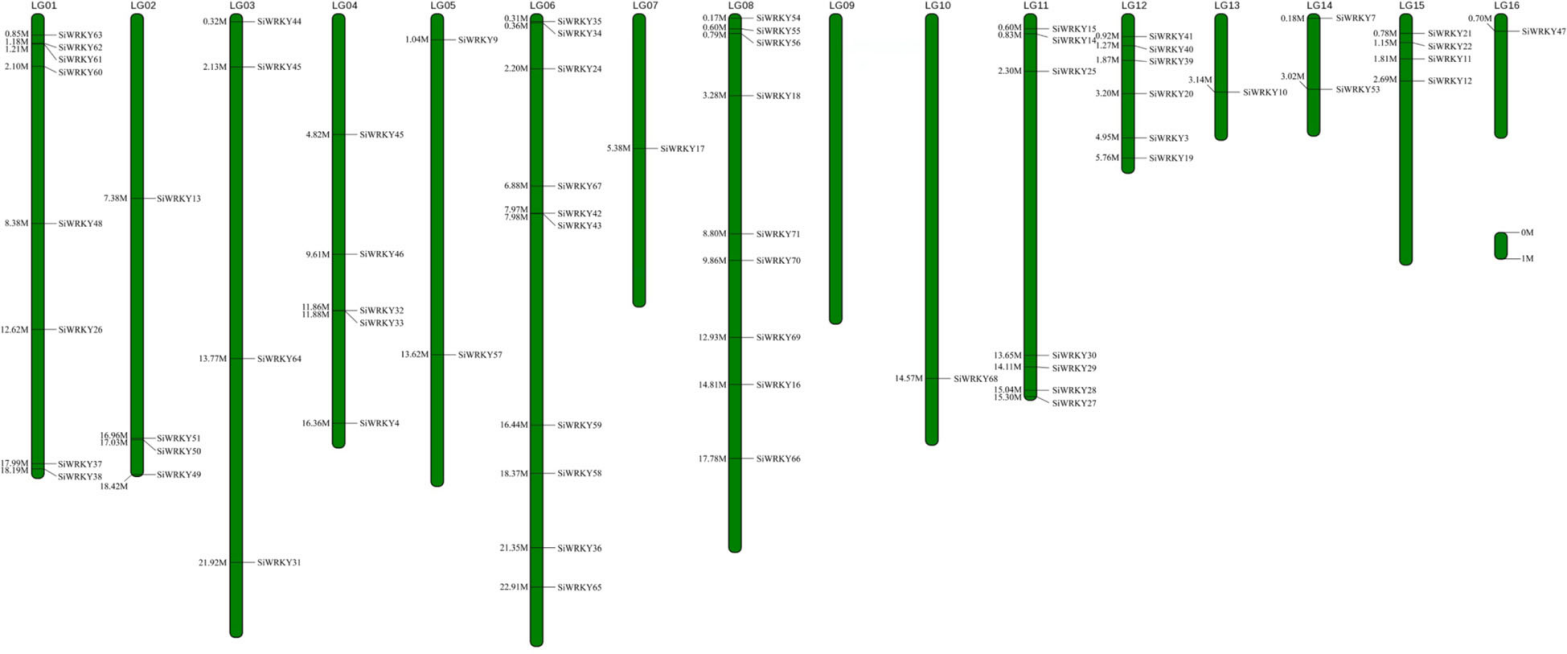
Distribution of *SiWRKY* genes within the sesame linkage group (LG). Vertical bars represent the LGs within the sesame genome. The LG number is indicated at the top of each LG. The scale on the right is in 1 million bases (Mb)

Of the 71 *SiWRKY* genes, 65 mapped to 15 sesame linkage groups (LGs), with the exception of LG09. Six genes (*SiWRKY1*, *2*, *5*, *6*, *8*, and *23*) mapped to unanchored scaffolds (Fig. 1, Table 1). LG06 contained the greatest number of sesame *WRKY* genes (10, 15.38%), whereas LG07, LG10, LG13, and LG16 contained only one gene each.

Syntenic analysis and comparison with the grapevine genome revealed that the sesame genome was duplicated in its entirety approximately 71 million years ago, creating two syntenic subgenomes [26]. Based on the synteny of these subgenomes, we identified 10 pairs of duplicated sesame *WRKY* genes (Additional file 1, Table 2). We were unable to identify any sesame *WRKY* genes using datasets for tandemly duplicated genes obtained from PTGBase [28], indicating that the *WRKY* gene family did not undergo tandem gene duplication; this finding is consistent with a previous report [29]. These results indicate that the *WRKY* gene family underwent whole genome duplication, without tandem gene duplication events (Table 2).

**Table 2:** Genome-wide duplication of *SiWRKY* genes

*Grapevine*	Subgenome1	Subgenome2
GSVIVT01008046001	*SiWRKY16*	*SiWRKY45*
GSVIVT01010525001	*SiWRKY69*	*SiWRKY49*
GSVIVT01014854001	*SiWRKY7*	*SiWRKY39*
GSVIVT01021397001	*SiWRKY55*	*SiWRKY3*
GSVIVT01026965001	*SiWRKY37*	*SiWRKY14*
GSVIVT01027069001	*SiWRKY38*	*SiWRKY15*
GSVIVT01030258001	*SiWRKY28*	*SiWRKY9*
GSVIVT01033063001	*SiWRKY11*	*SiWRKY10*
GSVIVT01033188001	*SiWRKY63*	*SiWRKY58*
GSVIVT01035426001	*SiWRKY60*	*SiWRKY59*

### Classification and phylogenetic analysis of the *SiWRKY* genes

We performed multiple sequence alignments to examine the structural features of each *SiWRKY* protein (Additional file 2). The results showed that 69 *SiWRKY* proteins contained one or two identical WRKYGQK domains. Although the WRKYGQK domain is highly conserved in WRKY proteins, *SiWRKY59* and *SiWRKY60* differed at one residue, with a glutamine being replaced by a lysine residue; this change is also found in *WRKYs* from tomato, *Arabidopsis*, and other plant species [13, 15, 19, 22]. Additionally, most of the *SiWRKY* proteins contained the C-X_4–7_-C-X_23_-H motif that forms the C_2_H_2_/C_2_HC-type zinc-finger structure.

**Fig. 2:**
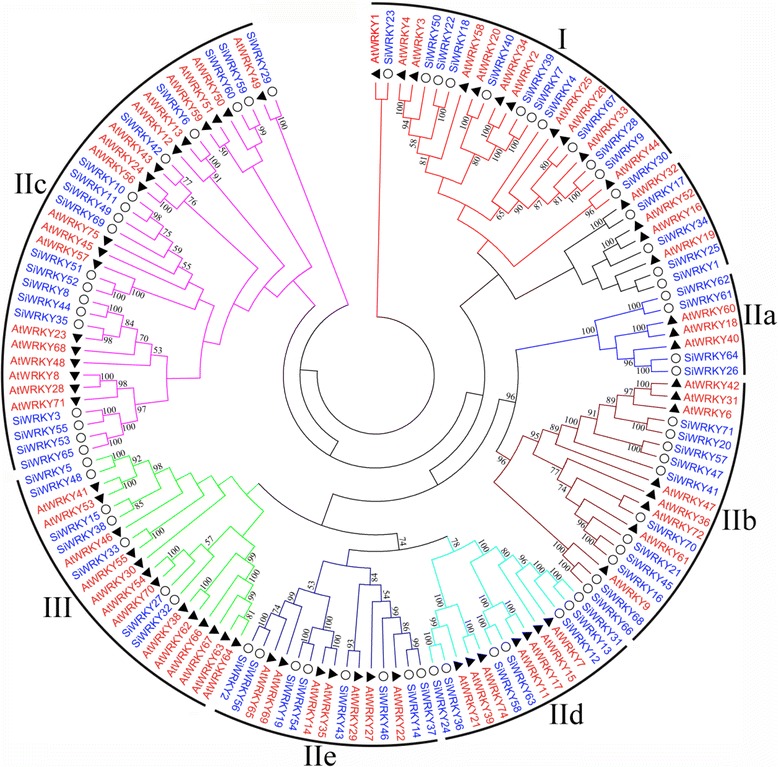
Phylogenetic analysis of the WRKY proteins in sesame and *Arabidopsis.* Multiple sequence alignments of WRKY amino-acid sequences were performed using ClustalX, and the phylogenetic tree was constructed using MEGA5 by the neighbor-joining (NJ) method and 1000 bootstrap replicates. The tree was divided into seven phylogenetic subgroups, designated I, IIa–e, and III. The bootstrap values were ≥50%

A phylogenetic tree was constructed using the neighbor-joining (NJ) method and based on multiple alignments of sesame and *Arabidopsis* WRKY domain aa sequences [15]. As shown in Fig. 2, the 71 *SiWRKYs* were classified into three groups (I, II, and III), and the *WRKYs *in Group II were further subdivided into five subgroups (IIa–e). Groups I, II, and III consisted of 12, 48, and seven *SiWRKY* proteins, respectively. A total of four, 11, 18, seven, and eight proteins were assigned to subgroups IIa, IIb, IIc, IId, and IIe, respectively.

### Conserved motifs and structure of the *SiWRKY* family genes

**Fig. 3:**
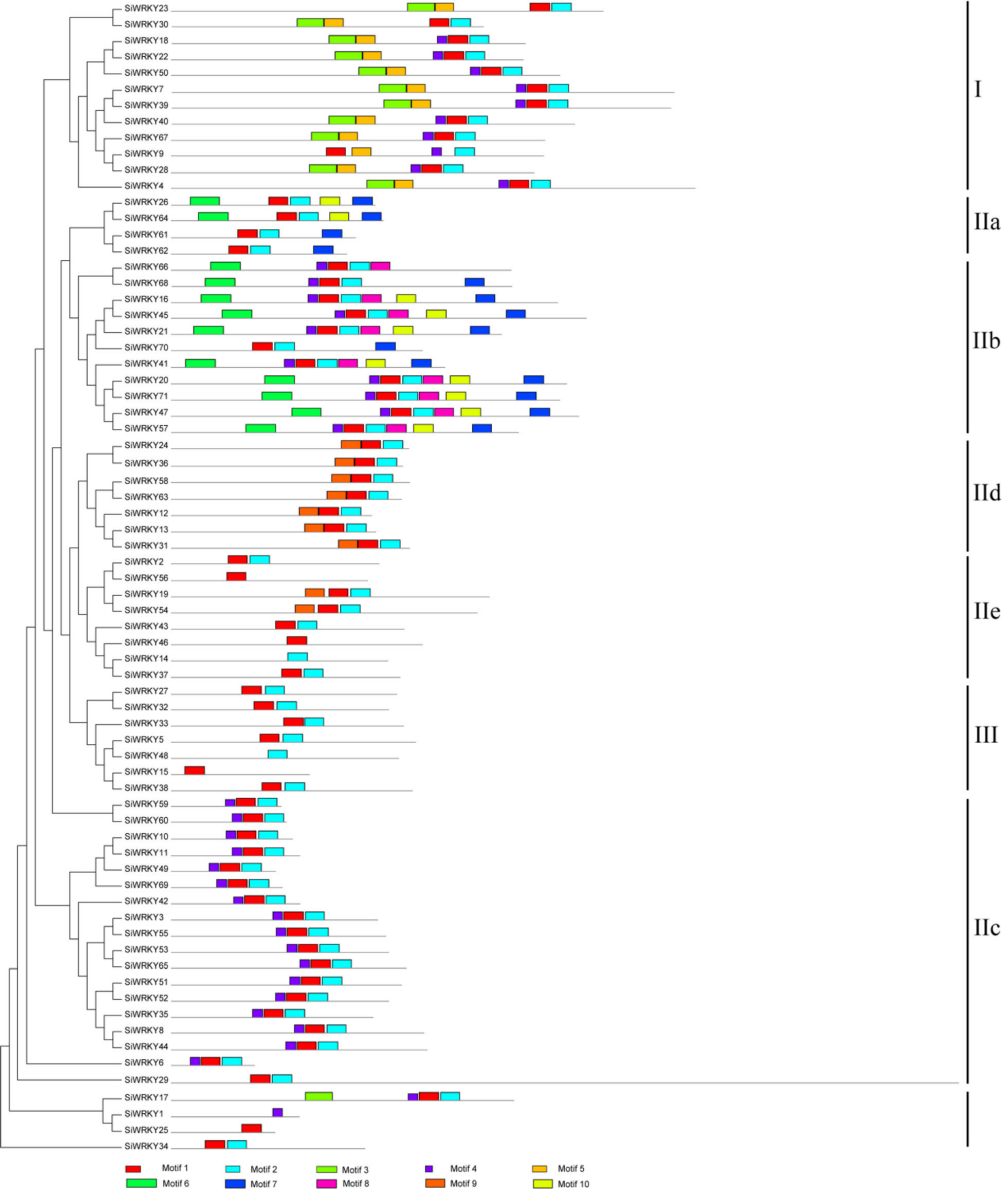
Conserved motifs of the *SiWRKY* proteins arranged according to their phylogenetic relationships. The NJ tree was constructed from the amino-acid sequences of sesame *WRKYs* using ClustalX and MEGA5 with 1000 bootstrap replicates. The conserved motifs in the *SiWRKY* proteins were identified using Multiple Em for Motif Elicitation (MEME). In total, 10 motifs were identified and are shown in different colors. Motif locations are also indicated

**Fig. 4:**
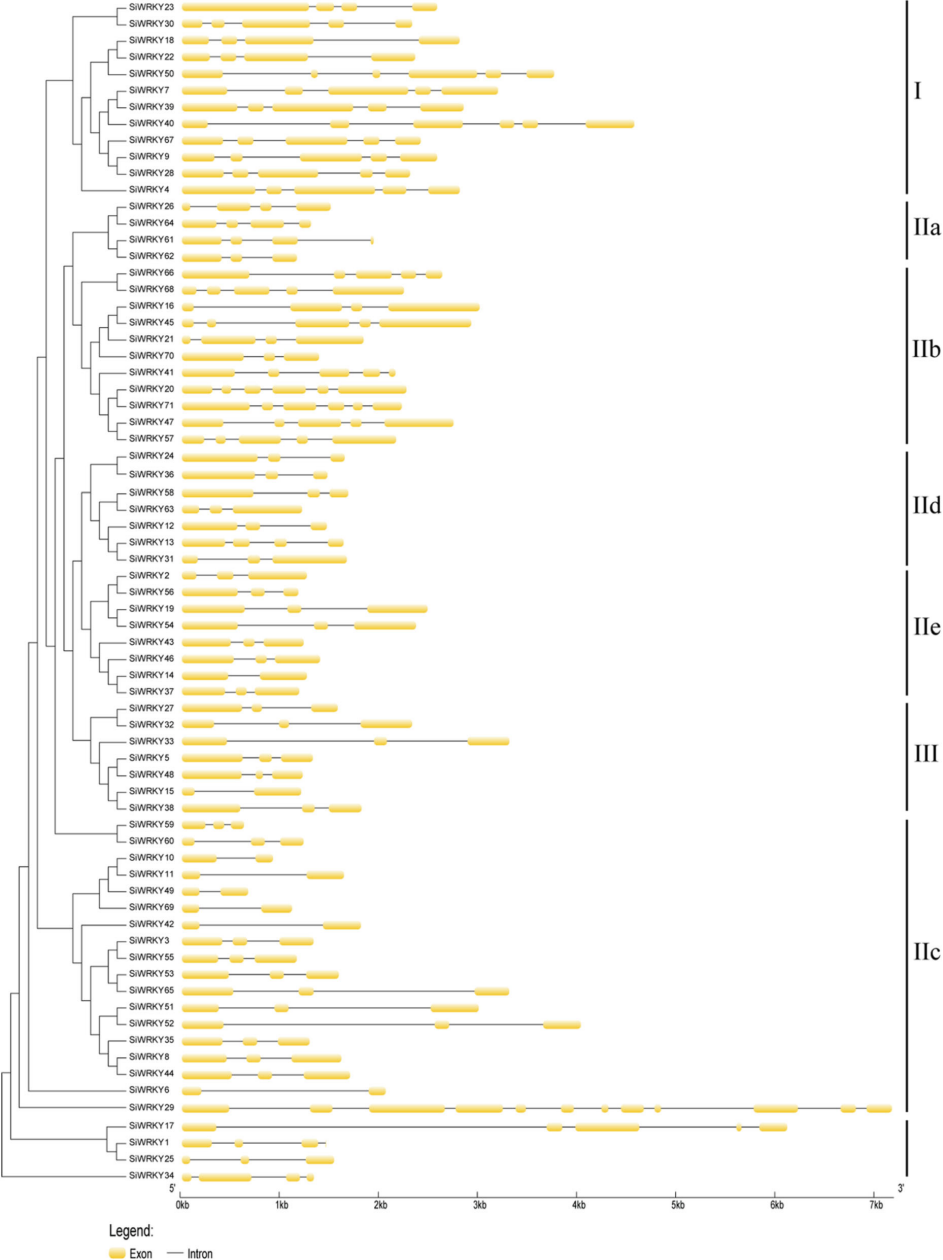
Structures of the 71 *SiWRKY* genes arranged in families. The NJ tree was constructed from the amino-acid sequences of the sesame *WRKYs* using ClustalX and MEGA5 with 1000 bootstrap replicates. Structural analyses of the *SiWRKY* genes were performed using the gene structure display server. The exons and introns are represented by colored boxes and black lines, respectively

Using the *SiWRKY* phylogenetic relationships data, we identified structural features of the sesame *WRKYs*, including conserved motifs and the locations of exons and introns. Using Multiple Em for Motif Elicitation (MEME) and InterPro Scan 5, we identified 10 conserved motifs in the sesame *WRKYs* (Fig. 3, Additional file 3) [30]. Motifs 1 and 4 were annotated as WRKY DNA-binding motifs, which is the fundamental characteristic of WRKY proteins. The motif 4 region sequence is conserved in N-terminal WRKY domains. All *SiWRKYs* contained at least one of these motifs, indicating the existence of features conserved in the *WRKY* gene family among the sesame WRKYs identified in this study. Group I proteins had two WRKY domains, each consisting of the conserved aa sequence WRKYGQK and a novel zinc-finger-like motif [15]. Group I might include the original genes from the other groups [30]. The gene structure predictions (Fig. 4) revealed that the *SiWRKY* genes had between one (*SiWRKY14*, *15*, *10*, *11*, *49*, *69*, *42*, and *6*) and 11 (*SiWRKY29*) introns.

### Tissue-specific expression profiling of the *SiWRKY* genes

**Fig. 5:**
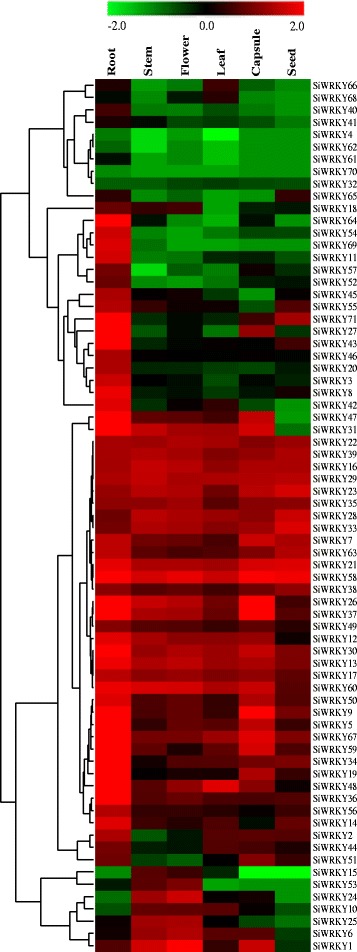
Expression profile analysis of *SiWRKY* genes in different sesame tissues. Transcriptome data (Reads Per Kilobase per Million mapped reads; RPKM) were used to measure the expression levels of *SiWRKY* genes in roots, stem, flowers, leaves, capsules, and seeds. The colored scale for the different expression levels is shown

To generate expression profiles of the *SiWRKY* genes under normal conditions, RNA sequence transcriptome data were collected and analyzed. The expression levels of the 71 *SiWRKY* genes were obtained on the basis of the reads per kilobase of transcript per million mapped reads (RPKM) values from six tissue samples (roots, stem, flowers, leaves, capsules, and seeds). The RPKM values of the transcripts were clustered hierarchically and displayed in a heat map (Fig. 5).

Quantification of transcript levels expressed in different tissues can be useful in determining gene function. The *SiWRKY* genes displayed diverse expression patterns, possibly reflecting the distinct roles of the different gene family members. A total of 66.20% (47/71), 30.99% (22/71), 32.39% (23/71), 21.13% (15/71), 43.66% (31/71), and 21.13% (15/71) of the *SiWRKY* genes were highly expressed (values >1) in roots, stem, flowers, leaves, capsules, and seeds, respectively. Most of the *SiWRKY* genes were expressed in all tissues, although *SiWRKY4*, *SiWRKY32*, *SiWRKY61*, *SiWRKY62*, and *SiWRKY70* were only expressed at low levels. Additionally, six *SiWRKY* genes (*SiWRKY22* and *SiWRKY39* in Group I, *SiWRKY16* and *SiWRKY21* in Group IIb, *SiWRKY58* in Group IId, and *SiWRKY29* in Group IIc) were continuously expressed at high levels (values >1) in all six organs, suggesting that these genes may be important for plant growth and organ development.

### Expression patterns of *SiWRKY*s in response to waterlogging and drought stresses

**Fig. 6:**
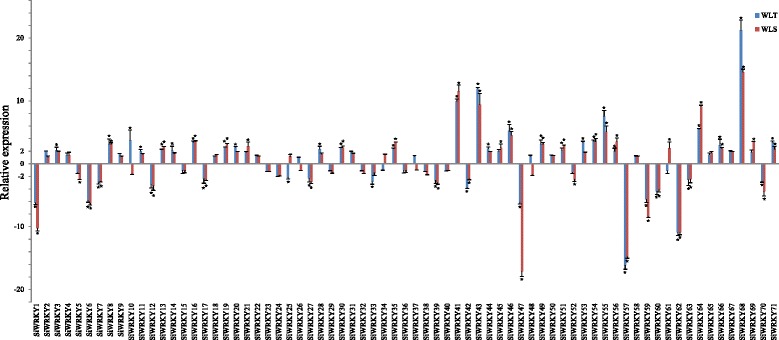
*SiWRKY* gene expression in sesame roots treated for 8 h with waterlogging stress compared with untreated controls. Transcript abundance was quantified using quantitative real-time polymerase chain reaction (qRT-PCR) and expression levels were normalized using sesame *β*-actin (SIN_1009011) as a reference gene. The mean expression levels from three independent biological replicates were analyzed for significance using *t*-tests (*p* < 0.01). The histograms represent the relative expression levels and rates of gene induction (stress/control). An asterisk (*) indicates a significant (2-fold) increase in gene expression in plants treated with waterlogging stress compared with untreated controls

The expression of *WRKY* genes has been examined under different stress conditions, including high salinity, drought, and high temperature; however, plant gene expression in response to waterlogging stress has not been studied extensively [13]. In this study, we investigated the expression of *SiWRKY* genes in the roots of sesame cultivars that were tolerant or sensitive to waterlogging stress using quantitative real-time polymerase chain reaction (qRT-PCR). As shown in Fig. 6, the majority of the *SiWRKY* genes (42 in the tolerant and 40 in the sensitive cultivar) were upregulated in both tolerant and sensitive waterlogged cultivars. Among these upregulated genes, >2-fold increases in expression (*P* < 0.01) were observed in 26 of the waterlogging-tolerant cultivars and 22 of the waterlogging-sensitive cultivars. Moreover, the same 18 *SiWRKY* genes (*SiWRKY8*, *SiWRKY13*, *SiWRKY16*, *SiWRKY19*, *SiWRKY30*, *SiWRKY35*, *SiWRKY41*, *SiWRKY43*, *SiWRKY46*, *SiWRKY49*, *SiWRKY51*, *SiWRKY54*, SiWRKY55, *SiWRKY56*, *SiWRKY64*, *SiWRKY66*, *SiWRKY68*, and *SiWRKY71*) displayed >2-fold increases in expression level in both waterlogging-tolerant and -sensitive cultivars. In addition, the *SiWRKY68* gene exhibited the highest expression level, with >10-fold increases in both waterlogging-tolerant and -sensitive cultivars. Waterlogging also decreased the transcript abundance of a large number of *SiWRKY* genes in roots. In total, 30 (42.3%) and 29 (40.8%) *SiWRKY* genes exhibited >2-fold downregulation (*P* < 0.01) in waterlogging-sensitive and -tolerant cultivars, respectively. In particular, the same 15 *SiWRKY* genes (*SiWRKY1*, *SiWRKY6*, *SiWRKY7*, *SiWRKY12*, *SiWRKY17*, *SiWRKY27*, *SiWRKY39*, *SiWRKY42*, *SiWRKY47*, *SiWRKY57*, *SiWRKY59*, *SiWRKY60*, *SiWRKY62*, *SiWRKY63*, and *SiWRKY70*) displayed exhibited >2-fold downregulation in both waterlogging-sensitive and -tolerant cultivars. Therefore, our results show differential expression (>2-fold upregulation or downregulation by a factor of two or more) of 33 genes in both waterlogging-sensitive and -tolerant cultivars, suggesting that these sesame genes play important roles in the response to waterlogging. Additionally, 27 of these 33 genes belong to *SiWRKY* gene Group II, while only three (*SiWRKY7*, *SiWRKY30*, and *SiWRKY39*), one (*SiWRKY27*), and two (*SiWRKY1* and *SiWRKY17*) belong to Group I, Group III, and the unknown group, respectively. Of the 18 upregulated genes, only *SiWRKY30* did not belong to Group II. In addition, 29 significant different expression *SiWRKYs* were found between waterlogging-tolerant and -sensitive cultivars, and 18 of them had high different expression level (>2 or <−2) (Additional file 4).

**Fig. 7:**
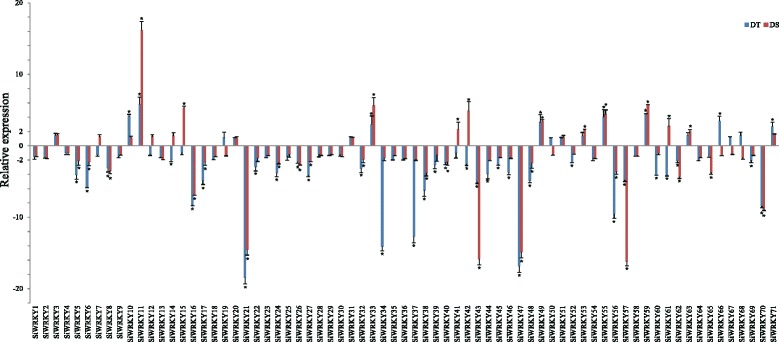
*SiWRKY* gene expression in sesame roots treated for 11 d with drought stress compared with untreated controls. Transcripts abundance was quantified using qRT-PCR and expression levels were normalized using sesame *β*-actin (SIN_1009011) as a reference gene. The mean expression levels from three independent biological replicates were analyzed for significance using *t*-tests (*p* < 0.01). The histograms represent the relative expression levels and rates of gene induction (stress/control). An asterisk (*) indicates a significant (2-fold) increase in gene expression in plants treated with drought stress compared with untreated controls

As shown in Fig. 7, drought stress decreased *SiWRKY* gene expression in sesame roots. Most of the *SiWRKY* genes were downregulated in both types of cultivars (53 drought-tolerant and 51 drought-sensitive cultivars). More genes were downregulated by >2-fold among the drought-tolerant (32 genes) than in the drought-sensitive (19 genes) cultivars. In contrast, 20 and 18 *SiWRKY* genes were upregulated in the drought-sensitive and drought-resistant sesame cultivars, respectively. The expression of five genes (*SiWRKY11*, *SiWRKY33*, *SiWRKY49*, *SiWRKY55*, and *SiWRKY59*) was upregulated by >2-fold and the expression of 19 genes (*SiWRKY5*, *SiWRKY6*, *SiWRKY8*, *SiWRKY16*, *SiWRKY17*, *SiWRKY21*, *SiWRKY24*, *SiWRKY26*, *SiWRKY27*, *SiWRKY32*, *SiWRKY38*, *SiWRKY40*, *SiWRKY43*, *SiWRKY47*, *SiWRKY48*, *SiWRKY56*, *SiWRKY57*, *SiWRKY62*, and *SiWRKY70*) was downregulated by >2-fold. Two *SiWRKY* genes (*SiWRKY42* and *SiWRKY61*) displayed increases in expression by >2-fold in the drought-sensitive cultivar, while decreases in expression by >2-fold were detected in the drought-tolerant cultivar. These 26 sesame genes displaying marked changes in expression might play important roles in drought stress responses. Eighteen of these genes belonged to Group II, one to Group I, six to Group III, and one to the unknown gene group. Additionaly, 33 significant different expression *SiWRKYs* were found between drought-tolerant and -sensitive cultivars, and 26 of them had high different expression level (>2 or <−2) (Additional file 5).

### Expression of selected *SiWRKY* genes in response to waterlogging and drought stresses

**Fig. 8:**
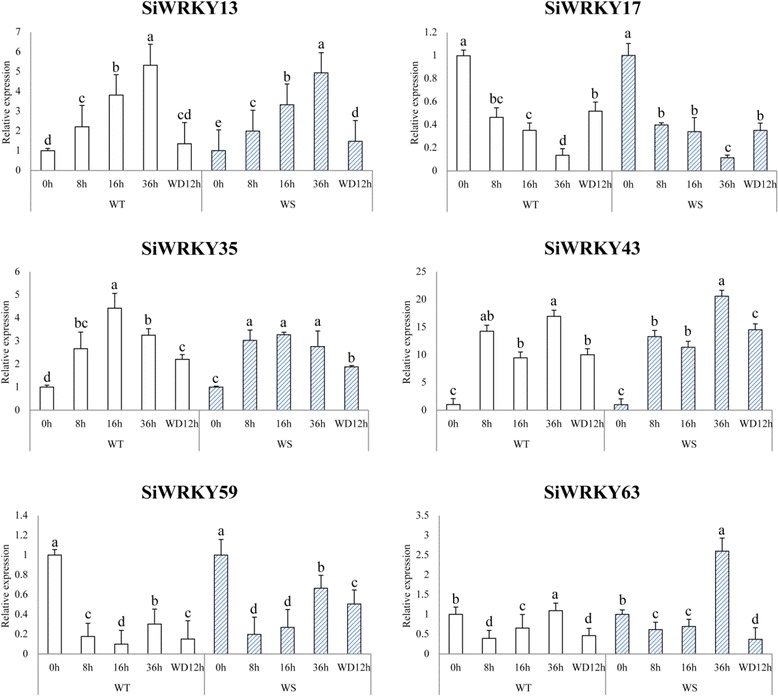
Expression profiles of six highly-expressed *SiWRKY* genes in response to waterlogging stress. The relative expression levels of six *SiWRKY* genes were measured from plants treated with waterlogging for 0, 8, 16, and 36 h, and also at 12 h after water was withdrawn (WD12h) from plants waterlogged for 36 h. The different columns represent different cultivars: a waterlogging-tolerant cultivar (WT) and a waterlogging-sensitive cultivar (WS). Three independent replicates were used to generate each expression value. The error bars represent standard deviations. Values with the same letter were not significantly different when assessed using Duncan’s multiple range test (*p* < 0.05, *n* = 3)

To confirm the identities of some of the genes important for waterlogging- and drought tolerance, 12 differential expression *SiWRKY* genes were selected and their expression levels quantified by qRT-PCR at different time-points after the onset of each abiotic stress. As shown in Fig. 8, six *SiWRKY* genes were expressed at different times following the start of the waterlogging treatment in both the waterlogging-tolerant and -sensitive cultivars (*P* < 0.05). The expression levels of *SiWRKY13*, *SiWRKY35*, and *SiWRKY43* increased during the waterlogging treatment, although the expression of each gene peaked at a different time. The peak expression of *SiWRKY35* occurred before that of *SiWRKY13* and *SiWRKY43*. In contrast, the expression of *SiWRKY17*, *SiWRKY59*, and *SiWRKY63* was downregulated by waterlogging. For the waterlogging -tolerant and –sensitive cultivars, the difference of the expression of these six genes mainly appeared at 36 h. We noticed that *SiWRKY35*, *SiWRKY43*, *SiWRKY59* and *SiWRKY63* expressed in a higher level in waterlogging sensitive cultivar than that in waterlogging tolerant cultivar, especially for the *SiWRKY63*. This result suggested that the different of the expression of *SiWRKY* genes might be one of the reasons for the tolerance of waterlogging for sesame varieties.

**Fig. 9:**
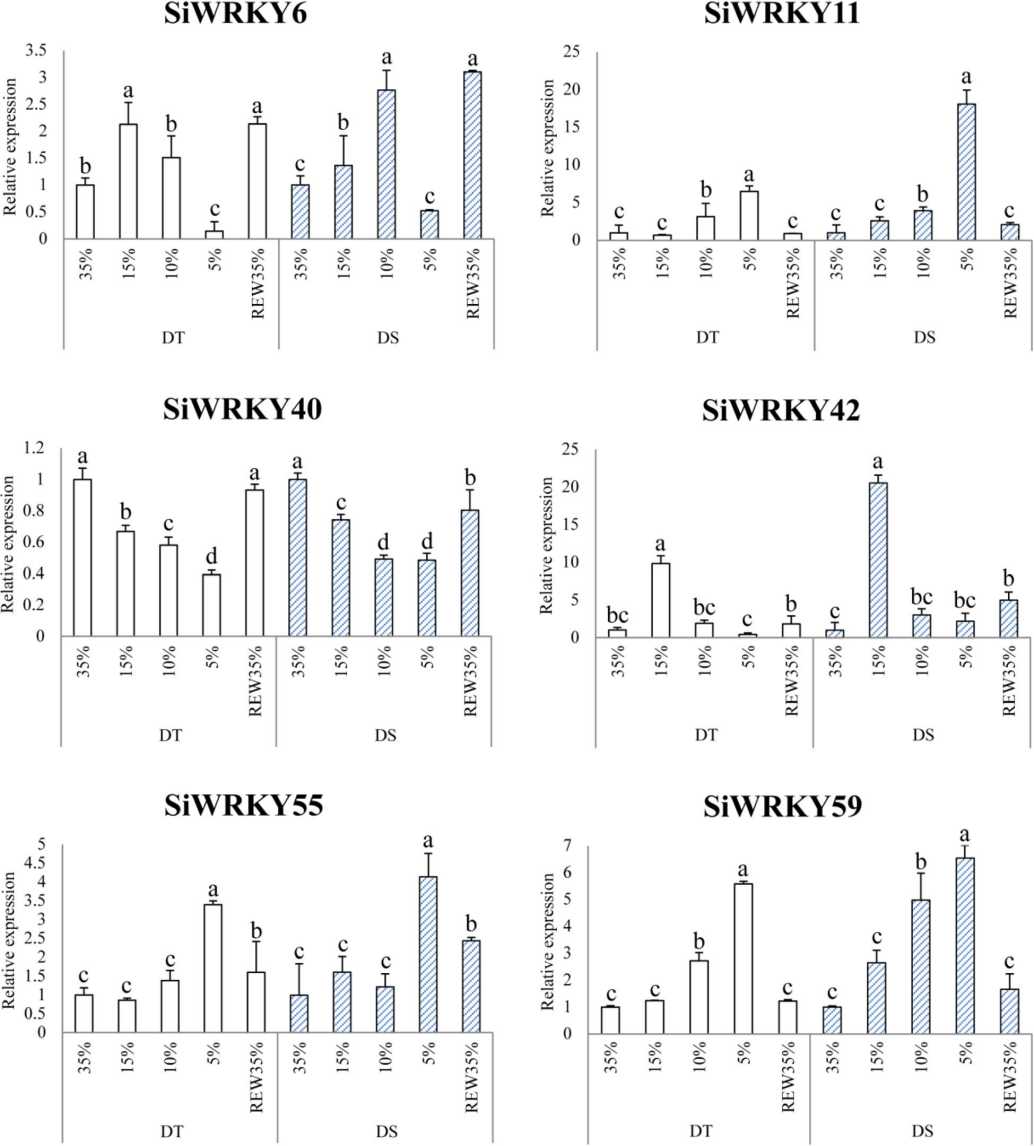
Expression profiles of six highly-expressed *SiWRKY* genes in response to drought stress. The relative expression levels of six *SiWRKY* genes were measured from plants treated with drought stress and harvested at a soil water content of 35%, 15%, 10%, 5%, and 35% after re-watering (REW35%). The different columns represent different cultivars: a drought-tolerant cultivar (DT) and a drought-sensitive cultivar (DS). Three independent replicates were used to generate each expression value. The error bars represent standard deviations. Values with the same letter were not significantly different when assessed using Duncan’s multiple range test (*p* < 0.05, *n* = 3)

As shown in Fig. 9, the expression of *SiWRKY* genes in response to drought differed significantly between drought-tolerant and -sensitive cultivars (*P* < 0.05). The expression of *SiWRKY6*, *SiWRKY11*, *SiWRKY42*, *SiWRKY55*, and *SiWRKY59* was considerably increased by drought stress. However, the expression of *SiWRKY6* was suppressed by severe drought conditions (5% soil water content) and recovered following re-watering (REW), indicating that severe drought decreases the expression of some sesame *WRKY* genes. Gene expression of *SiWRKY* under drought stress also showed significant different between drought- tolerant and - sensitive cultivars. Similar to the waterlogging stress, gene expression of most *SiWRKY* genes in the sensitive cultivar were higher than that in tolerant cultivar. The time-point that the largest difference of the gene expression appeared varied in each gene. For example, *SiWRKY11* had a much higher gene expression level in 5% soil water content of drought-tolerant cultivars, while *SiWRKY42* expressed highest in 15% soil water content.

## Discussion

### Number and type of sesame *WRKY* genes

The WRKY transcription factor family is one of the most important gene families involved in plant development and stress responses, and *WRKY* genes have been identified in many species, including *Arabidopsis*, rice, grape, maize, and cucumber [15, 20, 31–33]. Table 3 summarizes the numbers and types of *WRKY* genes found in higher plants, and illustrates their diversity among species that have had their genomes sequenced; the number of genes ranges from 55 in cucumber to 343 in rapeseed [34]. In this study, we identified 71 *WRKY* genes from a total of 27,148 annotated genes in the sesame genome. Relative to the genome size, the sesame *WRKY* gene family (350 Mb, 71 *WRKY* genes) is large compared with that of grape, cucumber, and castor bean. However, it is small compared with the *WRKY* gene families of *Arabidopsis* (107 Mb, 72 *WRKY* genes) and rice (440 Mb, 103 *WRKY* genes). Table 3 shows that one key difference between the sesame, *Arabidopsis*, and rice genomes is the number of Group III *WRKY* genes in each. The much more numerous Group III *WRKY* genes in *Arabidopsis* and rice is explained by tandem duplication and recent duplication events, which has led to a large-scale expansion of the gene families in these genomes [31, 35]. Recent gene duplication and tandem duplication events are the most important factors in the rapid expansion and evolution of gene families [35]. Previous research has demonstrated that the *Arabidopsis* Group III *WRKY* gene family expanded rapidly as a result of recent segmental- and tandem duplication events. Additionally, all of the tandemly duplicated *WRKY* genes in *Arabidopsis* belong to Group III, whereas we identified no segmentally or tandemly duplicated Group III *WRKY* genes in sesame. In this study, we identified 10 pairs of segmentally duplicated *SiWRKY* genes, but none of these belonged to Group III. Additionally, no *SiWRKY* genes had been generated by tandem duplication events in the sesame genome. Therefore, the small size of Group III in sesame is probably due to the absence of *WRKY* gene tandem duplication events.

**Table 3:** Numbers and types of *WRKY* genes in higher plants

Plant	Species	Genome size	Name	Total	Group							NG
					I	IIa	IIb	IIc	IId	IIe	III	
sesame	*Sesamum indicum*	350 Mb	*SiWRKY*	71	12	4	11	18	7	8	7	4
cucumber	*Cucumis sativus*	367 Mb	*CsWRKY*	55	10	4	4	16	8	7	6	0
*Arabidopsis*	*Arabidopsis thaliana*	107 Mb	*AtWRKY*	72	13	4	7	18	7	9	14	0
grape	*Vitis vinifera*	490 Mb	*VvWRKY*	59	12	3	8	15	7	6	6	2
rice	*Oryza sativa*	440 Mb	*OsWRKY*	103	15	4	8	15	7	11	36	0
tomato	*Solanum lycopersicum*	900 Mb	*SlWRKY*	78	15	5	8	16	6	17	11	3
flax	*Linum usitatissimum*	373 Mb	*LuWRKY*	97	24	4	13	16	11	12	15	2
soybean	*Glycine max*	1.1 Gb	*GmWRKY*	188	32	14	33	42	21	20	26	0
Castor bean	*Ricinus communis*	350 Mb	*CbWRKY*	47	9	3	10	12	3	5	5	0
*Brachypodium distachyon*	*Brachypodium distachyon*	272 Mb	*BdWRKY*	86	15	3	6	21	6	10	23	2
maize	*Zea mays*	2.3 Gb	*ZmWRKY*	136	27	7	11	29	14	17	31	0
cotton	*Gossypium raimondii*	761 Mb	*GrWRKY*	116	22	6	16	33	15	12	12	0
rapeseed	*Brassica napus*	630 Mb	*BnWRKY*	343	121	11	34	55	28	30	51	13
barley	*Hordeum vulgare*	5.1 Gb	*HvWRKY*	45	8	4	1	11	5	3	13	0
pear	*Pyrus bretschneideri*	527 Mb	*PbWRKY*	103	17	6	10	24	15	16	15	0

### Conserved motifs and structures of the sesame *WRKY* genes

Almost all of the *SiWRKYs* contained the WRKYGQK domain, although two Group IIc WRKYs (*SiWRKY59* and *SiWRKY60*) contained the WRKYGKK domain. This variant of the WRKY domain has also been found in pepper [9], tea [36], and apple proteins [13]. Waterlogging induced the expression of *SiWRKY59* and *SiWRKY60* (Fig. 6), indicating that these genes may be involved in sesame abiotic stress responses.

The conserved motifs and structural features of the sesame *WRKYs* were identified using MEME and InterPro Scan 5. The InterPro Scan 5 analysis suggested that *SiWRKY8* and *SiWRKY35* are PWRKY transcription factors, which represented a subset of Group IIc probable WRKY transcription factors from plants. PWRKY transcription factors, with the InterPro number IPR017396, were known to regulate various abiotic stress responses [37]. Thus, *SiWRKY8* and *SiWRKY35* might regulate the biotic and abiotic stress responses. This is consistent with the high expression of these genes in response to waterlogging. The *SiWRKY12*, *SiWRKY13*, *SiWRKY24*, *SiWRKY31*, *SiWRKY36*, *SiWRKY58*, and *SiWRKY63* genes contained Zn-cluster-domain sequences (IPR018872) and encoded WRKY-GCM1 zinc-finger-domain proteins, indicating that these genes acquired their functional diversity as developmental and regulatory genes [38]. *SiWRKY29* encodes an ATP-dependent metallopeptidase that belongs to the FtsH (IPR005936) protein family [39].

The exon/intron structural diversity found among the *SiWRKY* genes is related to their evolution [40]. An exon/intron distribution analysis demonstrated that *WRKY* genes from sesame had greater structural diversity than those from *Populus trichocarpa* or cassava. Most of the *SiWRKY* genes (33/71) had two introns, which is common in other plants, including *Pyrus bretschneideri* (59/103), *Populus trichocarpa* (49/104), cassava (42/85), and physic nut (30/58) [4, 21, 30, 41]. Most of the sesame MADS-box genes (63.2%, 36/57) have 0 or 1 intron, but most of *SiWRKY* genes (81.7%, 58/71) have 2 to 4 introns [25]. The sesame *WRKY* genes had significantly more introns than the sesame MADS-box genes, indicating that the WRKY gene structure in sesame is more complex.

### Diverse expression patterns of *SiWRKY* genes in different tissues

We analyzed the expression of *SiWRKY* genes in six different tissues. The results demonstrated variation in the expression patterns of *SiWRKY* genes. Most *SiWRKY* genes were highly expressed in roots, whereas a few *SiWRKY* genes were expressed in developing seeds. This is consistent with observations made in other plants, including rice [18], cucumber [33], grape [42], apple [13], cassava [30], cotton [43], physic nut [41], and cabbage [44]. Our results revealed that *SiWRKY* genes are expressed tissue-specifically and the high expression levels observed in roots might reflect their roles in responses to abiotic and biotic stresses that first affect plants below ground.

In total, 15 *SiWRKY* genes were highly expressed in at least five sesame tissues. Six of these genes (*SiWRKY16*, *SiWRKY21*, *SiWRKY22*, *SiWRKY29*, *SiWRKY39*, and *SiWRKY58*) were highly expressed in all sesame tissues. Highly expressed *WRKY* genes usually play important roles in plant development [45]. Therefore, we concluded that the 15 highly expressed *SiWRKY* genes might be important regulatory factors in sesame development, although further studies are required to verify the function of these genes. Most of these highly expressed *SiWRKY* genes belong to Groups I and IId. Previous research has demonstrated that Group I *WRKY* genes are ancestral to other *WRKY* genes in plants and are more likely to be constitutively expressed in different tissues [46]. For example, the Group I genes *SiWRKY28*, *SiWRKY29*, and *SiWRKY67* are expressed in most sesame tissues and highly expressed in response to waterlogging and drought stresses.

In contrast, 17 *SiWRKY* genes were expressed at low levels in all sesame tissues, and 19 *SiWRKY* genes were specifically expressed in only one tissue. Among the specifically expressed *SiWRKY* genes, *SiWRKY51* was expressed only in capsules and the remaining *SiWRKY* genes were expressed only in roots. These specifically or minimally expressed *SiWRKY* genes were found in all the *WRKY* gene subgroups, but many were found in Groups IIc and IIe. A number of Group IIc *WRKY* genes in *Arabidopsis* (e.g., *AtWRKY8*, *AtWRKY48*, *AtWRKY50*, and *AtWRKY57*) are involved in responses to bacterial and fungal pathogens, and in the jasmonic acid- and salicylic acid-mediated signaling pathways [27]. Therefore, although some Group IIc *SiWRKY* genes were expressed at low levels in most sesame tissues, they may play key roles in responses to biotic and abiotic stresses. In this study, *SiWRKY51* and *SiWRKY65* were highly expressed in the roots of waterlogged plants, whereas *SiWRKY10* and *SiWRKY53* were highly expressed in response to drought stress. These results indicate that some *SiWRKY* genes might only be expressed in response to particular abiotic stresses.

### Identification of *SiWRKY* genes involved in responses to abiotic stresses

Waterlogging and drought are the most serious abiotic stresses for sesame and result in significant losses (20%–50%) in sesame production within China [24, 47]. However, few abiotic stress tolerance genes have been identified in sesame. Recent research has demonstrated that *WRKY* genes are involved in responses to various stresses and there is now compelling evidence that *WRKYs* are plant transcription factors that regulate tolerance to abiotic stresses [48]. Gene expression studies have shown that 20 *AtWRKY* genes in *Arabidopsis*, 41 *OsWRKY* genes in rice, 66 *GmWRKY* genes in soybean, 41 *BrWRKY* genes in *Brassica rapa*, and 74 *BnWRKY* genes in rapeseed are involved in responses to abiotic stresses [14, 18, 34, 49–51]. In this study, 44 *SiWRKY* genes were expressed differentially in response to waterlogging and drought stresses, indicating that these genes may also be involved in responses to abiotic stresses. To identify the *WRKY* genes that regulate tolerance to abiotic stresses in sesame, waterlogging- and drought-tolerant and sensitive cultivars were investigated. As shown in Figs. 6 and 7, the expression of some *SiWRKY* genes differed significantly between the tolerant and sensitive sesame cultivars. For example, *SiWRKY10* was highly expressed in tolerant cultivars in response to waterlogging. Further analysis showed that responses to abiotic stresses occurred at different time-points. *SiWRKY17* and *SiWRKY59* were highly expressed after 8 h of waterlogging, whereas the expression of *SiWRKY13* and *SiWRKY43* peaked at 36 h after waterlogging began. This suggests that these *SiWRKY* genes might play important regulatory roles in sesame abiotic stress tolerance and may act at different stages of the stress response.

Compared with the *WRKY* genes that involved in the response of drought, cold and heat stresses, few *WRKY* genes that responded to waterlogging stress have been identified in plant. In addition, the expression pattern of *WRKY* genes under waterlogging stress was also unclear. Therefore, the expression of *SiWRKY* genes under waterlogging stress and the expression level of six highly expressed *SiWRKY* genes during the waterlogging treatment were detected in the present study. The qPCR results showed that 33 *SiWRKY* genes either increase or decrease their expression by a factor of two or more in both waterlogging tolerant and sensitive cultivars. With increasing treatment duration, both up-regulation and down-regulation of the *SiWRKY* genes were found. Previous studies have revealed that one *WRKY *gene could function in several disparate signaling pathways. For example, *AtWRKY70* functions in plant growth, drought response and cell death [52, 53]. Interestingly, we found that the *SiWRKY17* and *SiWRKY43* was induced to highly express under waterlogging stress, while the orthologous genes of them, *AtWRKY32* and *AtWRKY29*, was reported to respond to UV irradiation and heat stress, respectively. The result indicated that the orthologous *WRKY* genes might mediate different pathways and play different roles under the abiotic stress response in different species.

Both phylogeny-based and BLAST-based methods were used to identify *WRKY* gene orthologs in comparisons of sesame and *Arabidopsis*. A phylogenetic tree based on WRKY protein sequences from sesame and *Arabidopsis* was constructed and nodes with bootstrap values >50 were used to identify possible orthologs. In addition, standard BLASTP searches were applied to verify possible orthologs of *WRKY* genes in sesame and *Arabidopsis*, with relatively strict criteria (Additional file 6). In total, we found 10 orthologous pairs shared between sesame and *Arabidopsis* (Table 4). The functions of the 10 *AtWRKY* gene orthologs have been determined and all are involved in responses to abiotic stresses in *Arabidopsis*. *AtWRKY13* and *AtWRKY20* regulate tolerance to drought stress [54, 55] and their sesame orthologs, *SiWRKY6* and *SiWRKY40*, are also highly expressed in response to drought. Therefore, we conclude that the *SiWRKY* orthologs of *AtWRKY* genes also play key roles in the tolerance of abiotic stresses in sesame.

**Table 4:** Orthologous *WRKY* genes in sesame and *Arabidopsis*

Sesame	*Arabidopsis*	Function	Reference
*SiWRKY6*	*AtWRKY13*	Drought	[54]
*SiWRKY17*	*AtWRKY32*	UV irradiation, heavy metals	[62]
*SiWRKY29*	*AtWRKY49*	H_2_O_2_	[63]
*SiWRKY30*	*AtWRKY44*	drought	[64]
*SiWRKY33*	*AtWRKY55*	Oxidative stress	[65]
*SiWRKY35*	*AtWRKY23*	H_2_O_2_, ABA, mannitol	[66]
*SiWRKY40*	*AtWRKY20*	Drought	[55]
*SiWRKY42*	*AtWRKY43*	Nitrogen	[67]
*SiWRKY43*	*AtWRKY29*	Heat	[68]
*SiWRKY67*	*AtWRKY26*	Cold	[69]

### Functional divergence and segmental duplication of *WRKY* genes

Recent segmental duplication has occurred frequently in plant genomes because most plants are diploidized polyploids, and many duplicated chromosomal blocks have been retained [28]. Comparisons with grape suggest that the sesame genome underwent a recent genome duplication event, approximately 71 ± 19 million years ago [26]. In total, 10 pairs of sesame *WRKY* genes were identified as segmentally duplicated. In the *Arabidopsis* genome, the Group III *WRKY* genes are located in a recently segmentally duplicated region of the genome and are highly expressed in response to abiotic stresses. However, in this study, there was little correlation among the expression patterns of the duplicated sesame *WRKY* genes. For example, *SiWRKY16* was highly expressed in all sesame tissues, whereas the duplicated *SiWRKY45* gene was only expressed in roots. Additionally, the expression of *SiWRKY55* in response to drought was much higher than that of *SiWRKY3*.

Research using *Arabidopsis*, rice, and soybean has focused on identifying the gene targets of *WRKYs* and understanding the associated regulatory networks. One study showed that co-regulated networks involving *WRKY* genes were important in regulating the responses of pak-choi to a variety of abiotic stresses [27]. Wheat *TaWRKY19* regulates the expression of *DREB2A*, which encodes a key transcription factor that controls the expression of drought-related genes [56]. Therefore, the regulatory roles of *WRKY* genes in response to abiotic stresses are complex and further studies are required to understand their functions in sesame.

## Conclusions

In this study, we identified a total of 71 sesame *WRKY* genes and focused on those involved in responses to waterlogging and drought stresses. The distribution, classification, gene structure, and evolutionary characteristics of the sesame *WRKY* genes were investigated. The differential expression patterns of *SiWRKY* genes in the tissues of selected cultivars showed that these genes play different roles in sesame development and many exhibit tissue-specific expression patterns. Additionally, *SiWRKY* gene expression analyses revealed that some were markedly upregulated or downregulated in response to waterlogging and drought stresses. Our results also revealed significant differences in the abiotic-stress-induced expression of *WRKYs* in stress-sensitive and -tolerant sesame cultivars, indicating the involvement of these *WRKY* genes in abiotic stress tolerance in sesame. In conclusion, our study establishes a structural and functional framework to investigate sesame WRKY proteins. Although the sesame genome was sequenced several years ago, the identification of sesame abiotic-stress-related genes and investigations into their functions are still at an early stage. Our results will facilitate further studies into the functions of *WRKY* genes important in responses to abiotic stresses and the development of molecular breeding programs to enhance abiotic stress tolerance in sesame.

## Methods

### Identification of the *WRKY* gene family in sesame

All sesame protein sequences were obtained from the sesame genome database (http://ocri-genomics.org/Sinbase/) [26]. The *Arabidopsis thaliana AtWRKY* gene sequences were downloaded from UniProt (http://www.uniprot.org/). The HMM profile for the WRKY DNA-binding domain (PF03106) was downloaded from the PFAM protein families database (http://pfam.xfam.org) and used to identify *WRKY* genes from the sesame genome with HMMER 3.0 (http://hmmer.janelia.org/). BLAST analyses with all the *Arabidopsis WRKYs* were used to check the predicted *WRKYs* from the sesame database. The CDD (http://www.ncbi.nlm.nih.gov/cdd/) and PFAM databases (http://pfam.xfam.org/) were used to validate all the potential sesame *WRKY* genes identified by HMM and BLAST if they contained a WRKY domain. Multiple sequence alignments were used to confirm the conserved domains from the predicted WRKY sequences.

### Chromosomal location and phylogenetic analysis of the *WRKY* gene family in sesame

The physical positions of the *SiWRKY* genes were established using Sinbase (http://ocri-genomics.org/Sinbase/) and mapped to 16 LGs in the sesame genome using MapChart 2.3 [57]. Additionally, Clustal X 2.1 and MEGA 5.2 were used to construct a NJ phylogenetic tree based on the aa sequences of the sesame WRKY domains and selected *Arabidopsis WRKYs*, with 1000 bootstrap replicates. An alignment of sesame WRKY domains is shown in Additional file 1.

### Protein properties and sequence analysis

Protein MWs and isoelectric points (pIs) were predicted using the ProtParam program (ExPASy tools) based on their deduced aa sequences. The conserved motifs in the full-length WRKY proteins were identified using the MEME program (http://alternate.meme-suite.org/tools/meme). The parameters employed in the analysis were as follows: maximum number of motifs = 10; optimum width of motifs = 15–50 [30]. Additionally, all of the identified motifs were annotated using InterProScan (http://www.ebi.ac.uk/interpro/search/sequence-search). The exon/intron structures of the *SiWRKY* genes were determined by comparing their predicted coding sequence (CDS) with genomic sequences using the gene structure display server web-based bioinformatics tool (http://gsds.cbi.pku.edu.cn/) [58].

### Analysis of *SiWRKY* gene expression in different organs using transcriptomic data

Total RNA was extracted from roots, shoots, leaves, seed capsules, and seeds of Zhongzhi No. 13 grown under normal conditions. RNA pools were constructed using 3 μg of RNA per sample according to the manufacturer’s instructions and sequenced on a Gene Analyzer II system (Illumina, Inc., San Diego, CA, USA) according to the Illumina RNA-seq protocol. Gene expression levels were calculated in RPKM by taking into account the length of each gene and the read counts mapped. The sesame *WRKY* gene expression pattern analyses were performed using Gene Cluster 3.0, and the RPKM values for each gene in all tissue samples were log10 transformed. Finally, a heat map was generated using TreeView 1.0.4 [59].

### Plant materials and treatments

Waterlogging-tolerant (WT) cultivar 2541, waterlogging-sensitive (WS) cultivar 4508, drought-tolerant (DT) cultivar 0635, and drought-sensitive (DS) cultivar 4728 were all selected from sesame germplasm provided by the Oil Crops Research Institute, Chinese Academy of Agricultural Sciences, Wuhan, China.

For the waterlogging treatment, sesame plants at anthesis were irrigated until the soil surface was covered by a thin layer of water and this was maintained for 36 h. The plants were harvested 8 h later and their roots were immediately frozen in liquid nitrogen and stored at −80 °C prior to further analysis. Control plants were harvested 15 h before the waterlogging treatment. To further investigate waterlogging resistance-related genes, we harvested plants that had been waterlogged for 0, 8, 16, and 36 h, and also 12 h after water was withdrawn (WD12h) from plants waterlogged for 36 h. The roots were harvested as described previously.

For the drought stress treatment, water was withheld for 11 d from sesame plants at anthesis. Root samples were collected immediately thereafter and frozen in liquid nitrogen prior to analysis [24]. To further investigate genes important for drought resistance, we harvested plants when the soil water content was reached 35% (0 d controls), 15% (3 d), 10% (7 d), 5% (11 d), and 35% (14 d) after REW. The roots were harvested as described previously.

### qRT-PCR analyses of *SiWRKY* gene expression in response to waterlogging and drought stresses

RNA was extracted from the roots of each of the four cultivars using the EASYspin Plus Plant RNA Kit (Aidlab Biotechnologies, Beijing, China) [60] according to the manufacturer’s instructions. The RNA was quantified using a BIOMATE 3 spectrophotometer (Thermo Scientific, Worcester, MA, USA) and its integrity was confirmed using 1% agarose gel electrophoresis. A total of 1 mg of RNA was reverse-transcribed into cDNA using the iScript cDNA Synthesis kit (Bio-Rad, Hercules, CA, USA). A control amplicon was generated using the following primers for amplification of *β*-actin (SIN_1009011): forward primer, 5′-TTTGAGCAGGAACTGGACACT-3′, and reverse primer, 5′-ACAACACTTCTGGACAACGGA-3′. Gene expression levels were determined by performing qRT-PCR in triplicate on an Icycler iQ5 (Bio-Rad) using the SYBR Green Supermix kit (Bio-Rad), all according to the manufacturer’s instructions. Data were analyzed using iQ5 2.1 software (Bio-Rad) and the 2^–ΔΔCT^ method [61].

## Additional files

Additional file 1: Synteny of subgenomes in the sesame. The green bars represent the sesame chromosomes. The numbers 01–16 represent LGs within the sesame genome. Black lines on the green bars indicate the locations of sesame genes within the LGs. Colored lines indicate subgenomes in sesame (PDF 1332 kb)

Additional file 2: Alignment of *SiWRKY* domain aa sequences. The alignment was performed using Jalview. The conserved WRKY aa and zinc-finger motifs are highlighted in blue. Gaps are indicated by dashes. (PDF 506 kb)

Additional file 3: Conserved motifs of WRKY proteins in sesame. Significant motifs of more than 10 aa in length were predicted using MEME analysis. The motif IDs, consensus sequence lengths in aa, and *e*-value of each predicted motif are shown. (PDF 234 kb)

Additional file 4: The cultivar-specific *SiWRKY* gene expression in sesame roots treated for 8 h with waterlogging stress compared with untreated controls in cultivars. Transcript abundance was quantified using quantitative real-time polymerase chain reaction (qRT-PCR) and expression levels were normalized using sesame *β*-actin (SIN_1009011) as a reference gene. The mean expression levels from three independent biological replicates were analyzed for significance using *t*-tests (*p* < 0.01). The histograms represent the relative expression levels and rates of gene induction (stress/control). An asterisk (*) indicates a significant (2-fold) increase in gene expression in plants treated with waterlogging stress compared with untreated controls. (PDF 341 kb)

Additional file 5: The cultivar-specific *SiWRKY* gene expression in sesame roots treated for 11 d with drought stress compared with untreated controls in cultivars. Transcripts abundance was quantified using qRT-PCR and expression levels were normalized using sesame *β*-actin (SIN_1009011) as a reference gene. The mean expression levels from three independent biological replicates were analyzed for significance using *t*-tests (*p* < 0.01). The histograms represent the relative expression levels and rates of gene induction (stress/control). An asterisk (*) indicates a significant (2-fold) increase in gene expression in plants treated with drought stress compared with untreated controls. (PDF 544 kb)

Additional file 6: Synteny between *SiWRKY* genes in the sesame and *Arabidopsis* genomes. The green bars represent the chromosomes of the two species. The numbers 01–16 represent LGs within the sesame genome and the five *Arabidopsis* chromosomes are labeled Chr1–Chr5. Black lines on the green bars indicate the locations of *SiWRKY* genes on the chromosomes/within the LGs. Colored lines indicate orthologous genes in sesame and *Arabidopsis*. (PDF 425 kb)

## Abbreviations

Aa: amino acid(s)
ATP: Adenosine triphosphate
BLAST: Basic Local Alignment Search Tool
BLASTP: BLAST for protein sequences
CDD: Conserved domain database
CDS: Coding DNA sequence
DS: Drought-sensitive
DT: Drought-tolerant
FtsH: Filamentation temperature-sensitive H
GSDS: Gene structure display server
HMM: Hidden Markov model
LGs: Linkage groups
MEME: Multiple Em for Motif Elicitation
MW: Molecular weight
NJ: Neighbor-joining
pI: isoelectric point
qRT-PCR: quantitative real-time polymerase chain reaction
REW: After re-watering
RPKM: Reads per kilobase of transcript per million mapped reads
TD: Tandem duplication
WGD: Whole genome duplication
WS: Waterlogging-sensitive
WT: Waterlogging-tolerant
