# Supplementary figures and images for: Genome-wide analysis of WRKY gene family in the sesame genome and identification of the WRKY genes involved in responses to abiotic stresses
Source: BMC Plant Biol. 2017 Sep 11;17:152. doi: 10.1186/s12870-017-1099-y (PMC5594535; doi:10.1186/s12870-017-1099-y)

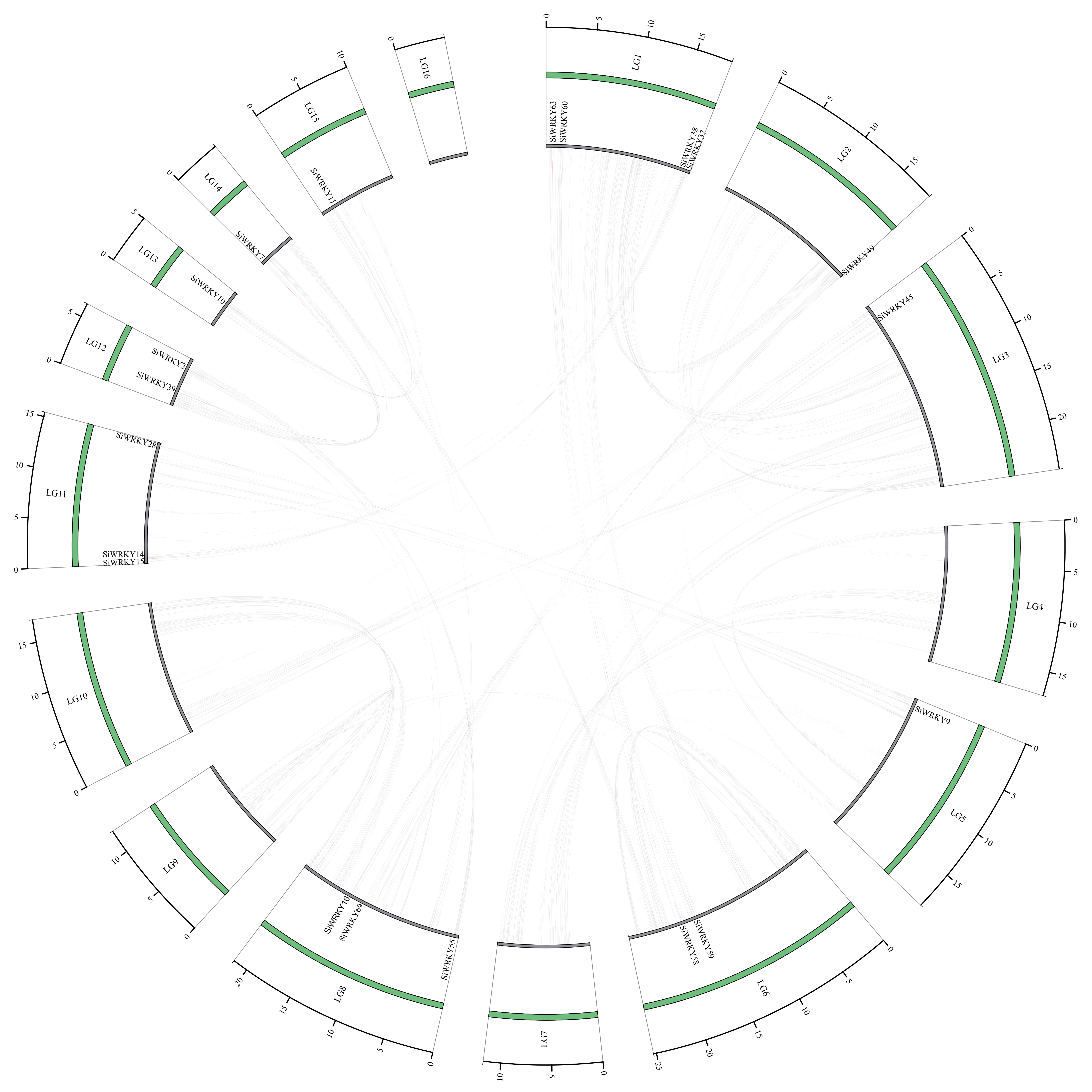

Supplement: Supplementary file 1 — Synteny of subgenomes in the sesame. The green bars represent the sesame chromosomes. The numbers 01–16 represent LGs within the sesame genome. Black lines on the green bars indicate the locations of sesame genes within the LGs. Colored lines indicate subgenomes in sesame (PDF 1332 kb) [file 12870_2017_1099_MOESM1_ESM.pdf]

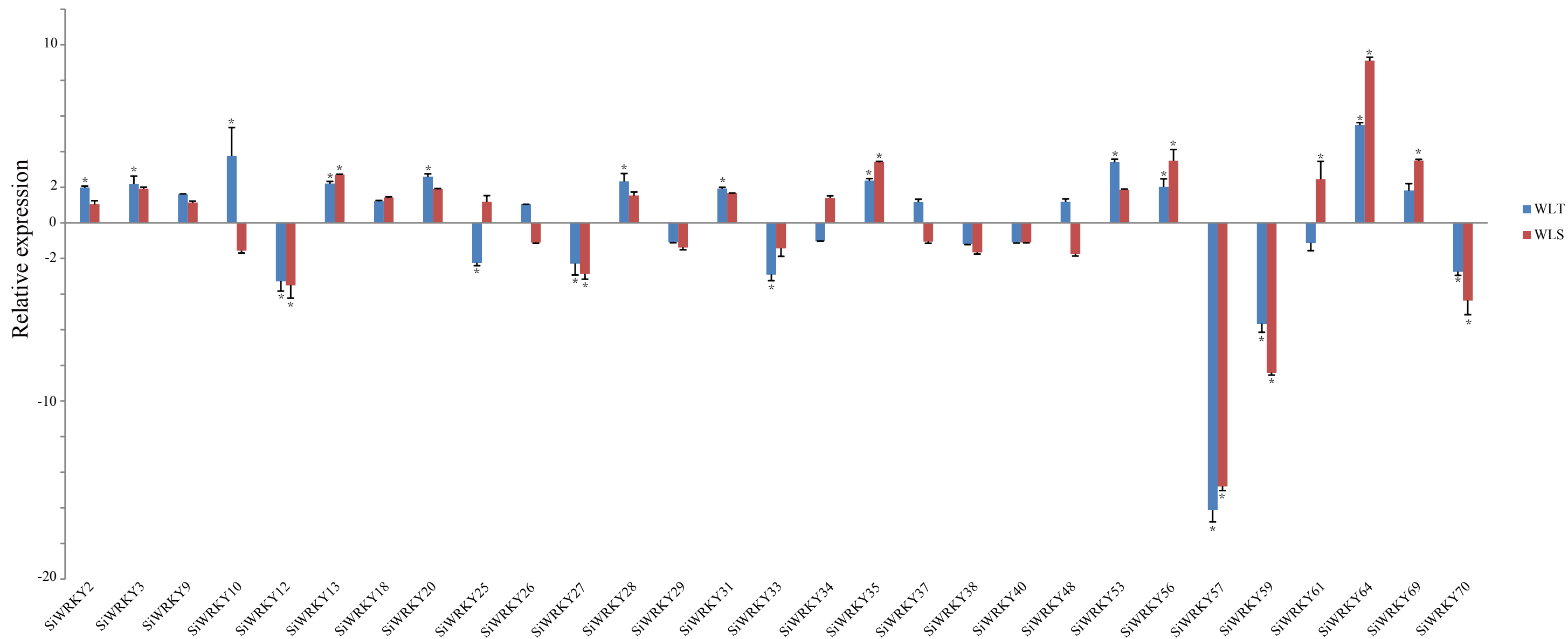

Supplement: Supplementary file 4 — The cultivar-specific SiWRKY gene expression in sesame roots treated for 8 h with waterlogging stress compared with untreated controls in cultivars. Transcript abundance was quantified using quantitative real-time polymerase chain reaction (qRT-PCR) and expression levels were normalized using sesame β-actin (SIN_1009011) as a reference gene. The mean expression levels from three independent biological replicates were analyzed for significance using t-tests (p < 0.01). The histograms represent the relative expression levels and rates of gene induction (stress/control). An asterisk (*) indicates a significant (2-fold) increase in gene expression in plants treated with waterlogging stress compared with untreated controls. (PDF 341 kb) [file 12870_2017_1099_MOESM4_ESM.pdf]

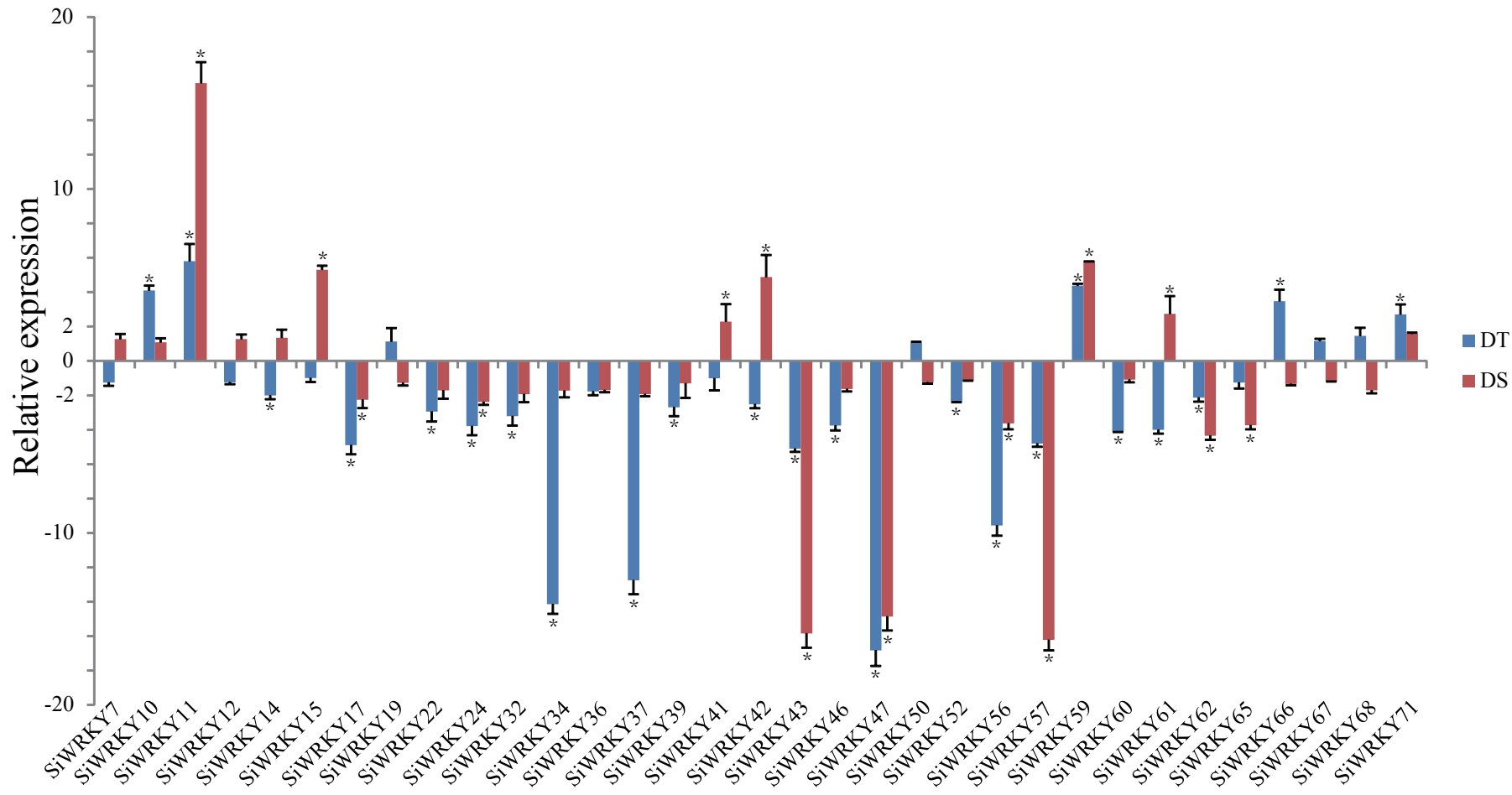

Supplement: Supplementary file 5 — The cultivar-specific SiWRKY gene expression in sesame roots treated for 11 d with drought stress compared with untreated controls in cultivars. Transcripts abundance was quantified using qRT-PCR and expression levels were normalized using sesame β-actin (SIN_1009011) as a reference gene. The mean expression levels from three independent biological replicates were analyzed for significance using t-tests (p < 0.01). The histograms represent the relative expression levels and rates of gene induction (stress/control). An asterisk (*) indicates a significant (2-fold) increase in gene expression in plants treated with drought stress compared with untreated controls. (PDF 544 kb) [file 12870_2017_1099_MOESM5_ESM.pdf]

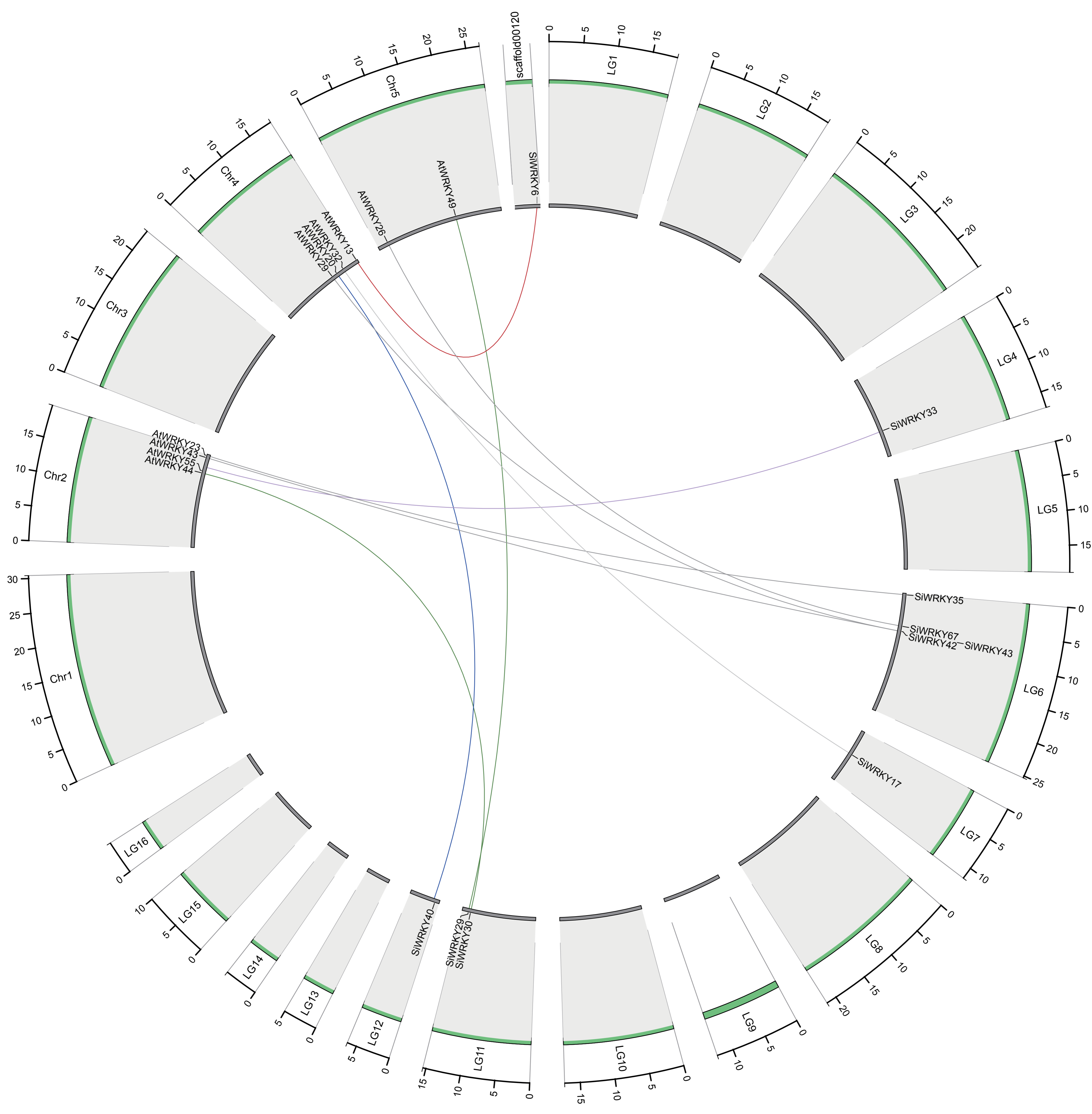

Supplement: Supplementary file 6 — Synteny between SiWRKY genes in the sesame and Arabidopsis genomes. The green bars represent the chromosomes of the two species. The numbers 01–16 represent LGs within the sesame genome and the five Arabidopsis chromosomes are labeled Chr1–Chr5. Black lines on the green bars indicate the locations of SiWRKY genes on the chromosomes/within the LGs. Colored lines indicate orthologous genes in sesame and Arabidopsis. (PDF 425 kb) [file 12870_2017_1099_MOESM6_ESM.pdf]
