# Supplementary material for: Genome-wide analysis of WRKY gene family in the sesame genome and identification of the WRKY genes involved in responses to abiotic stresses
Source: BMC Plant Biol. 2017 Sep 11;17:152. doi: 10.1186/s12870-017-1099-y (PMC5594535; doi:10.1186/s12870-017-1099-y)
Supplement: Supplementary file 3 — Conserved motifs of WRKY proteins in sesame. Significant motifs of more than 10 aa in length were predicted using MEME analysis. The motif IDs, consensus sequence lengths in aa, and e-value of each predicted motif are shown. (PDF 234 kb) [file 12870_2017_1099_MOESM3_ESM.pdf]

	Logo	E-value	Sites	Width
1.	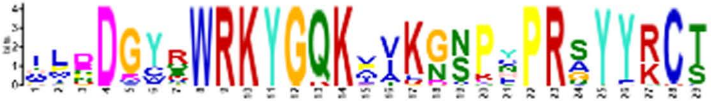	1.7e-1476	64	29
2.	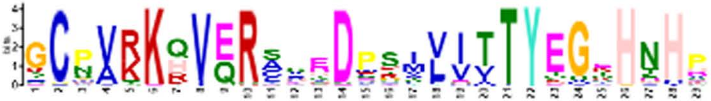	1.0e-1136	64	29
3.	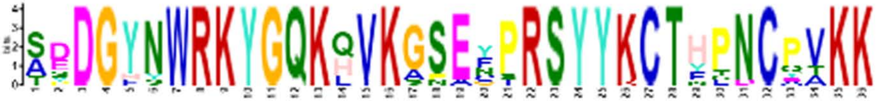	1.1e-284	12	36
4.	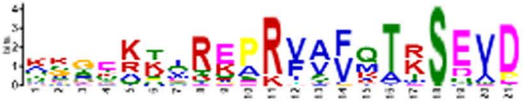	4.6e-201	34	21
5.	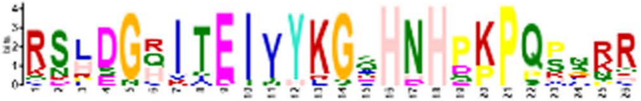	7.9e-130	13	26
6.	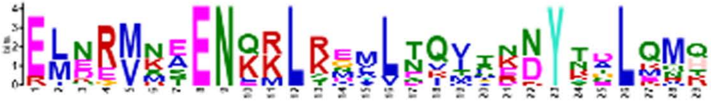	2.7e-122	14	29
7.	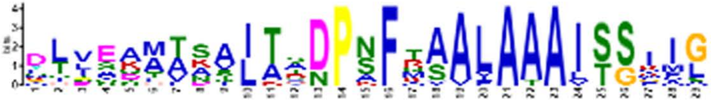	3.1e-107	14	29
8.	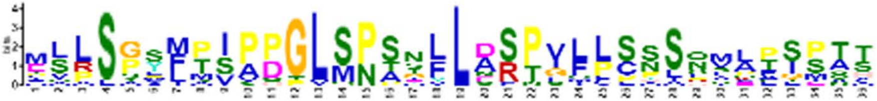	5.7e-088	15	36
9.	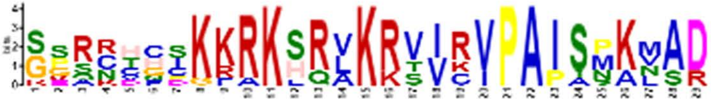	2.5e-068	9	29
10.	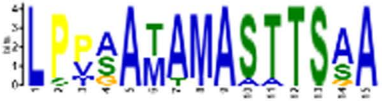	4.0e-057	11	15
